# Use of Near-Infrared Spectroscopic Analysis of Second Trimester Amniotic Fluid to Assess Preterm Births

**DOI:** 10.1155/2011/980985

**Published:** 2011-09-13

**Authors:** Kristin M. Power, Javier E. Sanchez-Galan, Gary W. Luskey, Kristine G. Koski, David H. Burns

**Affiliations:** ^1^Department of Chemistry, McGill University, Montreal, QC, Canada H3A 2K6; ^2^Division of Experimental Medicine, Faculty of Medicine, McGill University, Montreal, QC, Canada H3A 1A3; ^3^Division of Perinatal/Fetal Medicine, St. Mary's Hospital Center, Montreal, QC, Canada H3T 1H5; ^4^School of Dietetics and Human Nutrition, McGill University, Hacdonald Campus, Montreal, QC, Canada H9X 3V9

## Abstract

This pilot study investigated the possibility that metabolomic differences exist in second trimester of women delivering at term (≥37 weeks, *n* = 216) and preterm (≤35 weeks, *n* = 11). For this retrospective study, biobanked AF samples underwent near-infrared (NIR) spectral analysis using wavelengths from 700 to 1050 nm. Spectral data was compressed then optimized by multilinear regression to create a calibration model. The resultant model was able to classify term and preterm births based on differing AF metabolomic profiles with a sensitivity and specificity of 100%. When groups were classified using a prematurity index (PI), there was a statistical difference (*P* < 0.001) between the predicted preterm group (PI 0.77 ± 0.08) and the term group (PI 1.00 ± 0.02). In conclusion, the 2nd trimester AF samples showed distinct differences in metabolomic profiles between patients delivering preterm as compared to those at term in functional groups related to proteins, carbohydrates, fats, polyols, and water.

## 1. Introduction

Preterm birth, defined as birth prior to 37 weeks of gestation, is a leading cause of infant morbidity and mortality worldwide [1] and has been increasing [2, 3]. Since 1990, the number of preterm births has risen by 20% [4] and is even more significant when the increased costs associated with preterm pregnancies are considered [5]. However, despite advances in identifying some of the causes of preterm birth [6], our understanding of the physiologic process leading to preterm labor is poorly understood [7].

A growing body of literature has been examining possible proteomic markers for preterm labor and for intrauterine infection, a leading cause of preterm labor [6], in maternal serum [8, 9] and in amniotic fluid [9, 10]. None has produced significant positive predictive value for preterm births. On the other hand, metabolomics, which is the summation of ongoing cellular activity and downstream of protein metabolism [9, 11], may provide another interesting approach.

Metabolomics is the measurement of multiple small molecules in various tissues and fluids. These small molecules are the products of protein metabolism and cellular function within an organism. When examined as a whole, these metabolites can be viewed as biomarkers of a functional phenotype [12]. In the case of preterm labor, differences in metabolomic profiles found in amniotic fluid are thought to be possible since biological processes of the fetus and the mother both impact on its biochemical composition.

One successful approach to measure metabolomic profiles uses near-infrared (NIR) spectroscopy. The use of NIR vibrational spectroscopy preserves the matrix of constituent metabolites and provides important information about the interactions among the various constituents in situ. This can provide insight into metabolism, based on relational properties that cannot be captured when individual components are measured. Metabolite profiling using NIR spectroscopy has been used to detect disease in different scenarios where discrimination between groups is an objective [13]. Specifically, the application of NIR spectroscopy to amniotic fluid has been used to predict fetal lung maturity [14, 15]. Differences in these metabolomic profiles obtained by NIR spectroscopy also employ multivariate regression models and optimization functions [16, 17].

This pilot study was undertaken to test the hypothesis, using NIR spectroscopy, that differences in the metabolomic profile exist in second trimester amniotic fluid samples for term (≥37 weeks) compared to preterm births (≤35 weeks). We propose that identifying the existence of a metabolic fingerprint for preterm labor early in pregnancy could be of major importance in the appropriate ongoing monitoring of at-risk pregnancies and the development of a better understanding of the biologic basis of preterm labor.

## 2. Materials and Methods

This was a retrospective cohort study, approved by both the McGill Institutional Review Board and St. Mary's Hospital Center (Montreal, Canada). The population included 227 subjects recruited between 2000 and 2003 who provided a small volume of amniotic fluid for spectral profiling using NIR. Women were subdivided into term and preterm categories. Inclusion criteria for term births (*N* = 216) included age-related amniocentesis for genetic testing and a singleton pregnancy with no fetal complications. The preterm group included only patients with premature rupture of members (PROM) and/or preterm labour and a spontaneous vaginal delivery; patients who were induced or had a C-section were excluded. Demographics of the study participants are listed in Table 1. The two study groups were similar with respect to both maternal age and maternal BMI. The AF samples were obtained following genetic testing and stored at −80°C; there is minimal source of biochemical error resulting from repeated freezing and thawing of amniotic fluid [18, 19].

To determine the feasibility of estimating true premature births using spectral analysis of 2nd trimester amniotic fluid collected at 12–20 wks gestation, a calibration model was constructed using a set of AF samples with known birth outcomes in the NIR region of the spectra (700–1050 nm). This spectral region is known to contain functional group information on overtone bands of CH, NH, and OH moieties [20]. Glucose, proteins, fatty acids, oils, and myoglobin have been identified as contributing to the absorbance in this NIR region [21–23].

Prior to analysis, frozen AF samples were thawed at room temperature (25°C) for 30 minutes. NIR profiles were analyzed using a reflective spectrograph with a CCD detector (B&W TEK, Newark, DE) in randomized order. A flow sample cell with 10 mm path length was filled with 15 *μ*l of sample media. Spectra were recorded from 700–1050 nm at room temperature (25°C ± 1°C). The spectrophotometer was set to measure absorbance relative to air, and a signal average of 200 measurements with an integration time of 100 ms for each measurement was used. This measurement procedure involved rinsing the sample cell with 1 ml of 0.1 M NaOH followed by 5 ml of distilled, deionized water. The last injection of water was used to record a reference spectrum.

Quantification of the sample properties from NIR spectra consisted of determining the most parsimonious combination of variables in selected wavelength domains using a genetic algorithm optimization [16]. In this method preprocessing based on Haar wavelets, which is similar to jpeg compression of images, was used to objectively select wavelength regions. A combination of wavelength regions that most parsimoniously estimated prematurity index was determined by inverse least-squares regression, using a genetic algorithm optimization. The model investigated is of the form

(1) $$Y=\alpha_{0}+\alpha_{1}x_{1}+\alpha_{2}x_{2}+\cdots +\alpha_{n}x_{n},$$

where *Y* is the dependent variable or prematurity index (PI), *x*_1_, *x*_2_,…, *x*_*n*_ are independent variables (i.e., integrated wavelength region), and *α*_0_, *α*_1_,…, *α*_*n*_ are the coefficients determined from a set of calibration *x*'s. Many models were screened using the GA which is based on genetic principles such as mating, crossover, and mutation, to select the wavelength region that best separates the term and preterm groups [16].

Each sample was estimated independently using a leave-one-out cross-validation approach in a continuous multilinear model. For each individual in the population, the coefficients *α*_1_ to *α*_*n*_ of (1) were calculated by inverse least-squares regression using an independent calibration. Estimates of prematurity index were obtained by applying (1) with the determined *α*_*n*_ parameters and the *x*-values of a monitoring set. Fitness of the model for each wavelength region selected was calculated as the squared difference between the mean of the term and preterm groups divided by the sum of the pooled variance. A higher fitness corresponds to better separation between the two groups as calculated by a Student's *t*-test. Subsequent to the estimated prematurity index (PI) optimization, notched box plots, Student's *t*-test results, and a receiver operator curve (ROC) were used to separately determine the statistical characteristics of the groups. The optimum value for the sensitivity and specificity was determined from the ROC [24, 25].

Additionally, spectra were examined using the major chemical groups present in the 700–1050 nm region of the NIR spectra. Molecular absorbance regions in the NIR related to H_2_O, ROH, CH_2_, and NH_3_ were defined using known standards [20]. Means and standard deviations were then calculated for normal (≥37 wks) and preterm (≤35 wks) using these integrated regions. Likewise, ratios of pairs of selected functional groups (NH_3_/CH_2_, NH_3_/ROH, CH_2_/ROH CH_2_/ROH, NH_3_/H_2_O, and CH_2_/H_2_O) were determined to characterize concentration shifts in glucose, proteins fats and oils relative to water as well as polyols, which may play important roles in maintaining ATP concentrations, cellular redox potential, and in drawing water and solutes across the placenta [26].

All statistical analysis was done using the MATLAB (the Math Works Inc., MA) programming package.

## 3. Results

**Figure 1:**
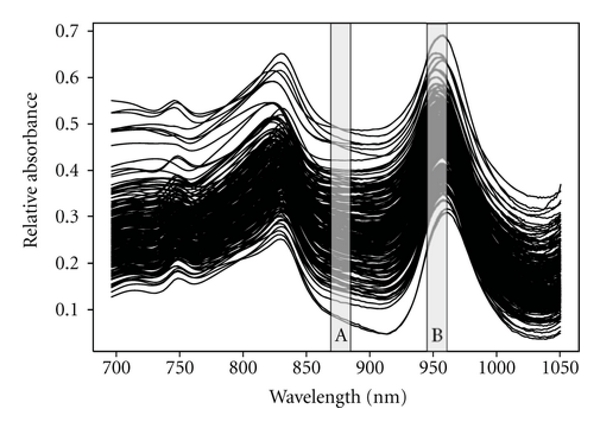
Raw data from 227 NIR spectral profiles of AF samples plotted relative to air. The boxes labeled A and B show the regions selected by a genetic algorithm, which give the best separation of term and preterm groups.

Population characteristics are described in Table 1. As expected, gestational age and birth weight differed.  Figure 1 describes NIR spectral differences for each individual in the range of 700–1050 nm. Of all of the possible wavelet combinations, only two wavelet regions were needed to develop the best model to distinguish preterm from the term births. Those wavelet regions selected by the genetic algorithm were at 872–879 nm (region A) and 943–954 nm (region B), respectively. The first wavelength region corresponded to third overtone CH_3_ and second overtone NH group vibrations [21]. This wavelet was negatively correlated with concentrations of chemicals absorbing in this region. Wavelet B selected an absorbance region characterized by aliphatic alcohol functional groups and third overtones from CH vibrations in the 943 to 954 nm wavelength range. These were negatively correlated with preterm births.

**Figure 2:**
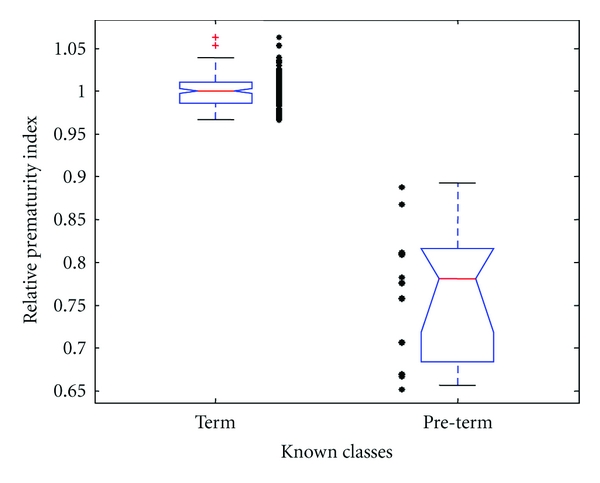
Box plots comparing the prematurity index for term and preterm births.

A parsimonious calibration model was constructed with the 2 wavelets selected by the genetic algorithm. Using these regions, the prematurity index (PI) was calculated. Each data point represented a blind estimation of the optimal separation value using the rest of the data for calibration. Results are represented in Figure 2 as notch box plots showing the statistical distribution for each group of classified samples [25]. A relative prematurity index (RPI) was calculated as a percentage of the median of the normals. The size of each box was determined by the quartile distribution about the median of the data. The median of the term group was 1.00 ± 0.02 and was 0.78 ± 0.08 for the preterm group. Notches of box plots, which do not overlap, have different medians at the 5% confidence level [25].

In addition, the mean and standard deviations of preterm and term prematurity indices were 0.77 ± 0.08 and 1.00 ± 0.02, respectively (*P* < 0.001).  Table 2 summarizes the results of the model's validation. Using only the 2 components for the calibration model, AF samples were classified into preterm and term groups with 100% sensitivity and specificity determined by a ROC curve. Positive and negative predictive values were 100% (Table 2).

**Figure 3:**
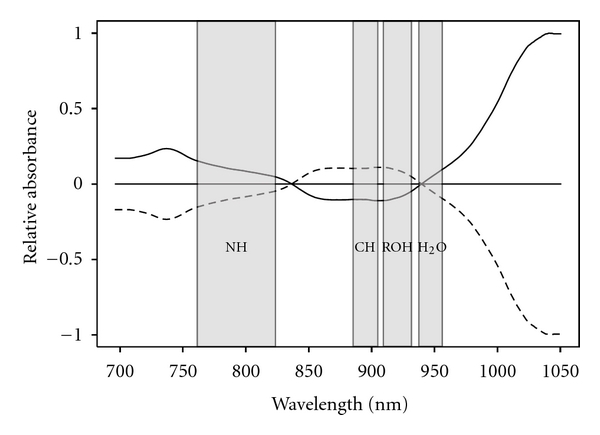
Normalized spectral absorbance for term and preterm groups. A solid line represents the term group (≥37 weeks), and the dashed line represents the pre-term group (≤35 weeks). This region of the spectrum consists of the 2nd and 3rd overtone absorption from CH, NH, and OH functional groups. Differing concentrations of proteins, fats, and carbohydrates in the matrix lead to increased or decreased absorbance in the spectral region corresponding to the functional groups. The result is essentially a metabolome fingerprint for preterm and term births using 2nd trimester amniotic fluid.

To further understand the relationship of the measured spectra to prematurity, differences in the amniotic fluid metabolomic profile between preterm and term infants arising from changes in selected spectral regions were examined. Regions selected for integration of the various functional groups as well as normalized spectra for the means for both the normal and preterm groups are shown in Figure 3. There were substantial metabolomic differences in the spectra between term and preterm infants. As well, ratios of the integrated signals from the different functional groups (Table 3) suggested nonsignificant trends in the concentrations of functional groups relative to water and/or polyols. There was a relative increase in concentration of protein as compared to carbohydrates and fats (NH/CH ratio) even though both decrease in total concentration as reflected in theNH/H_2_O and CH/H_2_Oratios. There was also nonsignificant increase in the NH/ROH ratio and a decrease in the CH/ROH ratio related to preterm births. 

## 4. Discussion

Previous studies have shown gestational length is difficult to predict [9]. Genomics, proteomics, mass spectroscopy, and other assays have been used for analysis of maternal and fetal biofluids with poor success rates [8, 9, 14, 15]. In contrast, the results of this pilot study showed that NIR spectral waveforms differed significantly in the range 700–1050 nm between term and preterm births. Varying concentrations of several functional groups contributed to the variation in these spectral analyses, confirming our hypothesis that metabolomic differences exist early in pregnancy in the amniotic fluid of second trimester pregnancies between subjects delivering preterm and those delivering at term.

Although the spectral features are broad, absorbance is still directly related to the concentration of CH, NH, and OH functional groups within the matrix. These differences seen in the preterm group corresponded to CH_3_ and NH (region A), aliphatic alcohols and CH functional groups (region B). Presence of proteins, water, and polyalcohols in AF are well known and can differ in concentration from sample to sample [26]. Our findings are intriguing in that they suggest the underlying processes ultimately leading to preterm delivery are present between 12 and 20 weeks of gestation. Prior studies have focused on identifying biomarkers present in amniotic fluid at the onset of preterm labor [8, 9, 14, 15]. Likewise, limitations such as AF samples taken after 22 weeks gestation, destructive analytical methods, and small populations plague many existing techniques [6, 9, 10, 14, 15]. Others have cited inability to distinguish preterm from term groups [10] and low positive predictive values [9]. 

Techniques from proteomics and genomics also have characterized few biomarkers in AF, thus limiting the amount of information about bioprocesses that might be associated with the amniotic fluid matrix [9–11]. The differing metabolomic profiles seen here in early pregnancy may represent an abnormal metabolic process in the fetus that ultimately predisposes the pregnancy to insults resulting in preterm labor and delivery. In addition, many recent technologies using proteomics and genomics rely on invasive procedures, expensive equipment, and technical expertise [6, 8–10]. The use of metabolomics with NIR spectroscopy is relatively inexpensive, easy to use and can characterized a metabolomic fingerprint resulting from multiple biological processes.

These results, however, should be considered preliminariy since the number of patients in the preterm delivery group is small. Despite this, our data using spectral analysis were able to distinguish between preterm and term deliveries with 100% sensitivity and specificity, which supports the potential diagnostic possibilities of this technique. Even though we were not able to identify specific differences in the ratios of the functional groups, probably owing to our small sample size, the strength of our results lies in the power of the spectral analysis to extract meaningful information about the metabolic profile from the 2nd trimester AF matrix that was associated with distinct metabolomic fingerprints early in pregnancy in our preterm and term deliveries. Moreover, the suggested metabolomic profiles were consistent with previous studies that show increased protein in amniotic fluid of premature infants [27], alterations in polyols in IUGR infants [26] and with a higher incidence of oligohydramnios [28]. 

Our results raise the question of whether obtaining metabolomic profiles of amniotic fluid in early pregnancy will help obstetricians identify those pregnancies destined to deliver preterm. Additionally, it may be possible to develop a noninvasive probe to analyze the NIR spectra of amniotic fluid on an ongoing basis. If future studies using larger sample sizes confirm these findings, this information would indicate that a “problematic metabolomic profile” emerges very early in pregnancy and could lead to much earlier identification of prematurity and earlier decision for potential therapies.

##  Conflict of Interests

None of the authors have any conflict of interests regarding this work.

## Figures and Tables

**Table 1:** Population Demographics.

Maternal/Fetal Characteristics^a^	≤35 weeks	≥37 weeks	*P*
Gestational Age (wks)	34.8 ± 2.4*	39.3 ± 1.9	N/A
Maternal Age (yrs)	37.5 ± 1.7	37.6 ± 2.4	0.4
Pre-pregnancy BMI (kg/m^2^)	23.8 ± 4.3	23.8 ± 4.9	0.6
Amniocentesis Week	15.5 ± 0.8^†^	15.2 ± 1.0^‡^	0.8
Birth weight (g)	2555 ± 540	3429 ± 623	<0.05
Parity	1.2 ± 0.6	1.1 ± 1.1	<0.05

^a^Data are reported as means ± standard deviation. Population characteristics for mothers delivering in term (*n *= 216) and preterm (*n *= 11). *Range is 28.5–35.1 weeks. ^†^Range is 14–17 weeks. ^‡^Range is 12–20 weeks.

**Table 2:** Results for the cross-validation of the calibration model and diagnostic statistics.

Preterm pregnancies		Control term pregnancies	
No. of group	11	No. of group	216
Prematurity index (PI) ($\overset{®}{\text{x}}\pm \mathrm{sd}\;$)	0.77 **± **0.08*	Prematurity index ($\overset{®}{\text{x}}\text{s}\pm \mathrm{sd}\;$)	1.00 **± **0.02
True positives	11	True negatives	216
False negatives	0	False positives	0
Sensitivity	100%	Specificity	100%
Positive predictive value	100%	Negative predictive value	100%

*P value < 0.001.

**Table 3:** Means and standard deviations by functional groups for term and preterm births^a^.

Functional group ratios	Gestation period^b^	
	Preterm < 35 weeks^c^	Term ≥ 37 weeks
NH/CH*	3.49 ± 0.5	3.43 ± 0.8
NH/ROH^†^	2.38 ± 0.3	2.36 ± 0.4
CH/ROH^‡^	0.69 ± 0.0	0.69 ± 0.0
NH/H_2_O^§^	2.28 ± 0.2	2.29 ± 0.2
CH/H_2_O^¶^	0.67 ± 0.1	0.69 ± 0.1

^a^Data are reported as means ± standard deviation. Birth outcomes and second trimester amniotic fluid functional groups ratios for mothers delivering in term (*n *= 216) and preterm (*n *= 10).

^b^All the differences between term and preterm groups were nonsignificant at a confidence level of 10%, with the exception of NH/CH which had a *P* value of 0.090.

^c^It is important to mention that one sample that had a gestational age of 35.1 was discarded as an outlier. Its exclusion from this ratio analysis was primarily because it had a gestational age 2 weeks above the mean and also was 500 g heavier than the average of the group. *Relative amount of protein to carbohydrates/fats. ^†^Relative amount of protein to modified polyalcohols.^‡^Relative amount of carbohydrates/fats to modified polyalcohols. ^§^Relative amount of protein to water. ^¶^Relative amount of carbohydrates/fats to water.

## References

[1] Engle WA (2006). A recommendation for the definition of “late preterm” (near-term) and the birth weight-gestational age classification system. *Seminars in Perinatology*.

[2] Lumley J (2003). Defining the problem: the epidemiology of preterm birth. *The British Journal of Obstetrics and Gynaecology*.

[3] Kramer MS, Platt R, Yang H (1998). Secular trends in preterm birth: a hospital-based cohort study. *Journal of the American Medical Association*.

[4] Hamilton BE, Martin JA, Ventura SJ (2006). Births: preliminary data for 2005. *National Vital Statistics Reports: From the Centers for Disease Control and Prevention, National Center for Health Statistics, National Vital Statistics System*.

[5] Wang ML, Dorer DJ, Fleming MP, Catlin EA (2004). Clinical outcomes of near-term infants. *Pediatrics*.

[6] El-Bastawissi AY, Williams MA, Riley DE, Hitti J, Krieger JN (2000). Amniotic fluid interleukin-6 and preterm delivery: a review. *Obstetrics and Gynecology*.

[7] Papatsonis DNM (2005). Prepregnancy counseling: preterm birth. *International Congress Series*.

[8] Shankar R, Gude N, Cullinane F, Brennecke S, Purcell AW, Moses EK (2005). An emerging role for comprehensive proteome analysis in human pregnancy research. *Reproduction*.

[9] Romero R, Espinoza J, Gotsch F (2006). The use of high-dimensional biology (genomics, transcriptomics, proteomics, and metabolomics) to understand the preterm parturition syndrome. *The British Journal of Obstetrics and Gynaecology*.

[10] Harrington PD, Vieira NE, Chen P (2006). Proteomic analysis of amniotic fluids using analysis of variance-principal component analysis and fuzzy rule-building expert systems applied to matrix-assisted laser desorption/ionization mass spectrometry. *Chemometrics and Intelligent Laboratory Systems*.

[11] Dunn WB, Ellis DI (2005). Metabolomics: current analytical platforms and methodologies. *Trends in Analytical Chemistry*.

[12] Seli E, Sakkas D, Scott R, Kwok SC, Rosendahl SM, Burns DH (2007). Noninvasive metabolomic profiling of embryo culture media using Raman and near-infrared spectroscopy correlates with reproductive potential of embryos in women undergoing in vitro fertilization. *Fertility and Sterility*.

[13] Ellis DI, Goodacre R (2006). Metabolic fingerprinting in disease diagnosis: biomedical applications of infrared and Raman spectroscopy. *The Analyst*.

[14] Liu KZ, Dembinski TC, Mantsch HH (1998). Rapid determination of fetal lung maturity from infrared spectra of amniotic fluid. *The American Journal of Obstetrics and Gynecology*.

[15] Liu KZ, Ahmed MK, Dembinski TC, Mantsch HH (1997). Prediction of fetal lung maturity from near-infrared spectra of amniotic fluid. *International Journal of Gynecology and Obstetrics*.

[16] Gributs CE, Burns DH (2006). Parsimonious calibration models for near-infrared spectroscopy using wavelets and scaling functions. *Chemometrics and Intelligent Laboratory Systems*.

[17] Turner E, Brewster JA, Simpson NA, Walker JJ, Fisher J (2007). Plasma from women with preeclampsia has a low lipid and ketone body content—a nuclear magnetic resonance study. *Hypertension in Pregnancy*.

[18] Keniston RC, Prescott GH, Pernoll ML (1975). Effects of freezing and thawing on certain properties of early gestation amniotic fluid. *Obstetrics and Gynecology*.

[19] Nelson TR, Gillies RJ, Powell DA, Schrader MC, Manchester DK, Pretorius DH (1987). High resolution of proton NMR spectroscopy of human amniotic fluid. *Prenatal Diagnosis*.

[20] Analytical Spectral Devices Inc. Near-IR Absorption Bands Chart. http://www.asdi.com/nir-chart_grid_rev-3.pdf.

[21] Gributs CEW, Burns DH (2006). In vivo near-infrared spectrometry. *Handbook of Vibrational Spectroscopy*.

[22] Greci LS, Gilson GJ, Nevils B, Izquierdo LA, Qualls CR, Curet LB (1998). Is amniotic fluid analysis the key to preterm labor? A model using interleukin-6 for predicting rapid delivery. *The American Journal of Obstetrics and Gynecology*.

[23] Romero R, Wu YK, Mazor M, Oyarzun E, Hobbins JC, Mitchell MD (1989). Amniotic fluid arachidonate lipoxygenase metabolites in preterm labor. *Prostaglandins, Leukotrienes and Essential Fatty Acids*.

[24] Zweig MH, Campbell G (1993). Receiver-operating characteristic (ROC) plots: a fundamental evaluation tool in clinical medicine. *Clinical Chemistry*.

[25] McGill R, Tukey JW, Larsen WA (1978). Variations of box plots. *The American Statistician*.

[26] Jauniaux E, Hempstock J, Teng C, Battaglia FC, Burton GJ (2005). Polyol concentrations in the fluid compartments of the human conceptus during the first trimester of pregnancy: maintenance of redox potential in a low oxygen environment. *The Journal of Clinical Endocrinology and Metabolism*.

[27] Bujold E, Romero R, Pedro J (2008). Proteomic profiling of amniotic fluid in preterm labor using two-dimensional liquid separation and mass spectrometry. *The Journal of Maternal-Fetal and Neonatal Medicine*.

[28] Magann EF, Doherty DA, Lutgendorf MA, Magann MI, Chauhan SP, Morrison JC (2010). Peripartum outcomes of high-risk pregnancies complicated by oligo- and polyhydramnios: a prospective longitudinal study. *The Journal of Obstetrics and Gynaecology Research*.

